# Identity-by-descent-based estimation of the X chromosome effective population size with application to sex-specific demographic history

**DOI:** 10.1093/g3journal/jkad165

**Published:** 2023-07-27

**Authors:** Ruoyi Cai, Brian L Browning, Sharon R Browning

**Affiliations:** Department of Biostatistics, University of Washington, Seattle, Washington, 98195, USA; Department of Biostatistics, University of Washington, Seattle, Washington, 98195, USA; Division of Medical Genetics, Department of Medicine, University of Washington, Seattle, Washington, 98195, USA; Department of Biostatistics, University of Washington, Seattle, Washington, 98195, USA

**Keywords:** identity by descent, effective population size, X chromosome

## Abstract

The effective size of a population ($N_{e}$) in the recent past can be estimated through analysis of identity-by-descent (IBD) segments. Several methods have been developed for estimating $N_{e}$ from autosomal IBD segments, but no such effort has been made with X chromosome IBD segments. In this work, we propose a method to estimate the X chromosome effective population size from X chromosome IBD segments. We show how to use the estimated autosome $N_{e}$ and X chromosome $N_{e}$ to estimate the female and male effective population sizes. We demonstrate the accuracy of our autosome and X chromosome $N_{e}$ estimation with simulated data. We find that the estimated female and male effective population sizes generally reflect the simulated sex-specific effective population sizes across the past 100 generations but that short-term differences between the estimated sex-specific $N_{e}$ across tens of generations may not reliably indicate true sex-specific differences. We analyzed the effective size of populations represented by samples of sequenced UK White British and UK Indian individuals from the UK Biobank.

## Teaser

Effective population size (Ne) in the recent past can be estimated through analysis of identity-by-descent (IBD) segments. In this work, Cai et al. propose a method to estimate the X chromosome effective population size from X chromosome IBD segments. The authors show how to use the estimated autosome Ne and X chromosome Ne to estimate female and male effective population sizes.

## Introduction

The effective size of a population ($N_{e}$) is defined as the number of breeding individuals in an idealized randomly mating population that has the same expected value of a parameter of interest as the actual population under consideration (Wright 1931). The effective population size is a fundamental parameter in population genetics because it determines the strength of genetic drift and the efficacy of evolutionary forces such as mutation, selection, and migration (Charlesworth 2009). Previous studies have demonstrated that estimates of the recent effective population size can reveal aspects of a population's demographic history, such as past population growth or bottleneck events (Browning and Browning 2015; Browning *et al.* 2018).

Identity-by-descent (IBD) segments can be used to estimate the effective population size in the recent past. IBD segments are haplotypes that 2 or more individuals have inherited from a common ancestor. IBD segments end at positions where crossovers have occurred in the meioses between the common ancestor and the descendant individuals. IBD segments for which the common ancestor is in the distant past tend to be shorter than IBD segments from a recent common ancestor because there are more meioses since the common ancestor on which crossovers can occur. The autosomes and the X chromosome are both subject to recombination, making them both amenable to IBD segment analysis (Buffalo *et al.* 2016; Henden *et al.* 2016). Previous studies have developed methods for estimating the recent effective population size from autosomal IBD segments (Palamara *et al.* 2012; Browning and Browning 2015; Browning *et al.* 2018). However, no such effort has been made with X chromosome IBD segments.

The autosomes are equally influenced by female and male demographic processes. In contrast, the X chromosome is influenced more strongly by female demographic processes than by male demographic processes. This is because females have 2 copies of the X chromosome, while males have only 1. Thus, a comparison of statistics from the X chromosome and from the autosome can be used to estimate sex-specific parameters such as the female and male effective population sizes (Hammer *et al.* 2008; Ségurel *et al.* 2008; Bustamante and Ramachandran 2009; Keinan *et al.* 2009; Bryc *et al.* 2010; Heyer *et al.* 2012; Goldberg and Rosenberg 2015; Buffalo *et al.* 2016; Shringarpure *et al.* 2016; Clemente *et al.* 2018; Musharoff *et al.* 2019).

The standard Wright–Fisher model used to define the effective population size assumes equal numbers of breeding females and males. To define the female and male effective population sizes, we consider an idealized Wright–Fisher population modified to allow for different numbers of females and males. The female and male effective sizes ($N_{e}^{f}$ and $N_{e}^{m}$) of a study population are the number of females and males in an idealized Wright–Fisher population that would give the same rates of IBD on autosomes and sex chromosomes as the study population (Wang *et al.* 2016).

In this work, we develop an IBD-based method to estimate the X chromosome effective population size in each generation in recent history. We show that the estimated X chromosome $N_{e}$ can be combined with the estimated autosome $N_{e}$ to estimate the female and male effective population sizes over time. This application of the X chromosome $N_{e}$ provides a useful complement to previous methods that give only a single time-averaged estimate for the sex-specific effective sizes in human populations (Hammer *et al.* 2008; Emery *et al.* 2010; Clemente *et al.* 2018). Through simulation studies, we validate the theoretical relationship between the autosome and X chromosome $N_{e}$, and we show that our method can accurately estimate the autosome and X chromosome $N_{e}$ in simulated populations. We investigate the application of the X chromosome $N_{e}$ to estimate the sex-specific $N_{e}$ and show that pronounced differences in the estimated female and male effective population sizes over many generations are reasonably accurate, but confidence intervals do not always cover the true values, and short-term observed differences in the estimated effective population sizes may not represent true differences. We use our method to infer the autosome and X chromosome $N_{e}$, as well as the sex-specific $N_{e}$, for several human populations.

## Methods

### Probability modeling for the X chromosome

All meioses from mothers transmit an X chromosome, while half of meioses from fathers transmit an X chromosome. Over many generations, approximately 2/3 of meioses from parents to offspring that transmit an X chromosome will be from females, as we show below. However, the actual proportion of meioses transmitting an X chromosome that are from females in recent generations depends on the proportion of females in these generations. For example, if the sample consists entirely of females (who receive an X chromosome from each parent), half of the meioses in the most recent generation that transmit an X chromosome are from females. If the sample is made up solely of males (who receive only 1 X chromosome, inherited from the mother), all the meioses that directly contribute the X chromosomes in the sample are from females. If half the sampled individuals are female and half are male, then 2/3 of the meioses in the previous generation that contribute the X chromosomes in the sample will be from females. We show in the next paragraph that when half of the samples are female, the expected value for this ratio will be 2/3 in all prior generations, regardless of sex-specific demographic forces.

Consider the lineage of a randomly selected haplotype at a point in the genome. Let $p_{g}$ be the probability that the ancestral haplotype at generation *g* before present is carried by a female, where g=0 corresponds to the generation of the sampled individuals, g=1 to the generation of their parents, and so on. A given haplotype carried by a female has a 50% probability that its parent haplotype is carried by a female, while a given haplotype carried by a male always has its parent haplotype carried by a female. Thus, for g≥0,


$$p_{g+1}=0.5p_{g}+(1-p_{g})=1-0.5p_{g}.$$

If $p_{g}=2/3\;$, then $p_{g+1}=2/3$. If the sampled haplotype is randomly chosen from a set of individuals with equal numbers of females and males, then the sampled haplotype has probability $p_{0}=2/3$ of being carried by a female, and as a result, $p_{g}=2/3$ for all *g*. More generally, it can be shown by mathematical induction that


$$p_{g}=\frac{2}{3}\left(1-{\left(-\frac{1}{2}\right)}^{g}\right)+{\left(-\frac{1}{2}\right)}^{g}p_{0},$$

which converges to 2/3 for large *g*. This equation is similar to equations for X chromosome admixture proportions and for X chromosome allele frequencies in a particular breeding system (Goldberg and Rosenberg 2015; Rosenberg 2016). In what follows, we assume that $p_{g}=2/3$ for all *g*.

Consider IBD sharing on the X chromosome resulting from a common ancestor living *g* generations ago. Let *F* be the number of female meioses out of the 2g meioses in the path of inheritance. Then, *F* follows a binomial(2g,2/3) distribution. Assuming Haldane’s (1919) model, the length of the IBD segment is exponentially distributed with rate 3F/2 per Morgan, as crossovers that end an IBD segment happen only in female meiosis on the X chromosome (which comprise 2/3 of the meioses), and the female recombination rate is 3/2 per Morgan on the X chromosome when using sex-averaged genetic distances. As an approximation, we model the probability distribution of the length of an X chromosome IBD segment resulting from a coalescence event occurring *g* generations ago as an exponential random variable with rate 2g per Morgan, which is the same model that we use for estimating the IBD-based effective population size in autosomal data (Browning and Browning 2015). We compared the simulated distribution of draws from an exponential(3F/2) distribution with *F* drawn from a binomial(2g,2/3) distribution to the exponential(2g) distribution for different values of *g* (Supplementary Fig. 1). The result shows that the exponential(2g) model approximates the distribution of IBD lengths on the X chromosome very well for g>1.

### IBD-based estimation of the X chromosome effective population size

Our method for IBD-based estimation of the X chromosome effective population size history is based on IBDNe, which was designed to estimate the recent effective population size from autosomal IBD segments (Browning and Browning 2015). The IBDNe method calculates the expected length distribution of IBD segments exceeding a given length threshold (2 cM [centiMorgans] by default) for a given effective population size history. It finds the effective population size history that equates the observed and expected IBD length distributions using an iterative scheme. IBDNe applies smoothing over intervals of 8 generations to avoid overfitting. Although IBDNe was designed for autosomal data, we show that it can also be used with X chromosome data, with some adjustments to the analysis procedure that we describe in the following paragraphs.

The first adjustment ensures proper inference of IBD segments on the X chromosome by encoding male X chromosome genotypes as haploid. Male X chromosome genotypes are frequently coded as homozygous diploid genotypes rather than haploid genotypes, which typically results in duplicate reported IBD segments when using IBD detection methods designed for autosomal data. The hap-ibd program (Zhou *et al.* 2020) correctly analyzes chromosome X data with haploid male genotypes. Alternatively, if males are coded as homozygous diploid on the X chromosome, we can exclude IBD pairs involving the second haplotype of males after running hap-ibd on the diploid-coded data. We exclude the pseudoautosomal regions and regions outside the genetic map from the analysis.

The second adjustment is to use a sex-averaged genetic map, as we also do for the autosomes. X chromosomes transmitted from females are subject to recombination, while X chromosomes transmitted from males are not (except for the pseudoautosomal regions, which we exclude from all analyses). Genetic maps, such as the HapMap map (International HapMap Consortium 2007) and the deCODE map (Halldorsson *et al.* 2019), typically report the female-specific recombination map for the X chromosome. Since an average of 2/3 of meioses transmitting an X chromosome are from females, the sex-averaged X chromosome recombination map can be obtained by multiplying female-specific genetic distances by 2/3. For example, a region with length 3 cM on the female-specific map has length 2 cM on the sex-averaged map. Equivalently, the sex-averaged recombination rates can be obtained by multiplying female-specific recombination rates by 2/3. For example, an X chromosome region with a female-specific recombination rate of $3\times 10^{-8}$ per base pair per generation has a sex-averaged recombination rate of $2\times 10^{-8}$ per base pair per generation.

The third adjustment ensures equal numbers of sampled females and males. If the sample is unbalanced, we remove some randomly selected females or males to obtain equal numbers of females and males. Consequently, $p_{0}$, the proportion of sampled X chromosome haplotypes carried by females, is 2/3, and hence $p_{g}$, the probability that the ancestral haplotype of a sampled X chromosome haplotype at *g* generations before present is carried by a female, is always 2/3 (see *Probability modeling for the X chromosome*).

The fourth adjustment modifies the IBDNe “npairs” parameter to be equal to the number of analyzed haplotype pairs. By default, IBDNe assumes that each individual contributes 2 haplotypes to the analysis and that all cross-individual pairs are analyzed, resulting in (2n)(2n−2)/2 haplotype pairs when there are *n* individuals. On the X chromosome, with $n_{f}$ females and $n_{m}$ males, the number of haplotype pairs is


(1) $$2n_{f}(2n_{f}-2)/2+n_{m}(n_{m}-1)/2+2n_{f}n_{m}.$$

We set the IBDNe “npairs” parameter to the value in Equation 1 when analyzing X chromosome data after adjusting it for removal of close relative pairs as described below.

The fifth adjustment is to manually remove detected IBD segments corresponding to close relatives (parent–offspring and siblings). By default, IBDNe identifies the close relatives using the input IBD segments, removes the IBD segments between them, and adjusts the “npairs” parameter to account for the removed sample pairs. However, this strategy does not work for the X chromosome because one cannot reliably detect close relatives using only X chromosome data. We thus turn off IBDNe's filtering of close relatives by setting “filtersamples = false.” We can identify close relatives based on autosomal data or from a pedigree file if available and then remove IBD segments for these pairs and update the “npairs” parameter accordingly in the chromosome X analysis. Removing only IBD segments between the related pairs rather than completely removing 1 individual from each pair of relatives reduces the loss of information from the data.

The sixth adjustment enables calculation of confidence intervals. IBDNe obtains confidence intervals for the estimated effective population sizes by bootstrapping over chromosomes. We thus divide the X chromosome into 6 pieces of equal cM length and treat these as separate “chromosomes” in the analysis with IBDNe.

### From the X chromosome effective population size to the sex-specific effective population size

We next describe how the estimated X chromosome effective population size can be used in conjunction with the estimated autosome effective population size to estimate the female and male effective population sizes. We will write $N_{g}^{X}$ and $N_{g}^{A}$ for the X chromosome and autosomal effective population sizes at generation *g*. And we will write $N_{g}^{f}$ and $N_{g}^{m}$ for the female and male effective population sizes at generation *g*, which can be derived from the X chromosome and autosomal effective population sizes as described below.

The IBD-based effective population size (for autosomes or X chromosomes) is defined in terms of the conditional coalescence probability for a Wright–Fisher population. We first consider autosomes. For a randomly selected pair of haplotypes, let *G* be the number of generations before present that the haplotypes coalesce. Conditional on the haplotypes not coalescing by generation g−1 before present, the ancestral haplotypes are distinct at that generation. For them to coalesce at generation *g* before present, their 2 parental haplotypes at generation g must be the same haplotype. If the diploid autosomal effective population size is $N_{g}^{A}$ at *g* generations before present, there are $2N_{g}^{A}$ autosomal haplotypes available, and the probability that the 2 parental autosomal haplotypes are the same is thus $1/(2N_{g}^{A})$. That is, $P_{A}(G=g|G>g-1)=1/(2N_{g}^{A})$. Thus, if we know the value of $P_{A}(G=g|G>g-1)$, then we can obtain the effective population size at *g* generations before present:


$$N_{g}^{A}=1/(2P_{A}(G=g|G>g-1)).$$

On the X chromosome, the conditional coalescence probability can be obtained by considering that 2/3 of meioses are from female parents, while 1/3 are from male parents. For coalescence to occur, both haplotypes’ parent haplotypes must be the same. This means both haplotypes must be inherited from parents that have the same sex, and the second haplotype must have the same parent haplotype as that of the first (within that sex). Thus,


$$P_{X}(G=g|G>g-1)=\frac{{\left(2/3\right)}^{2}}{2N_{g}^{f}}+\frac{{\left(1/3\right)}^{2}}{N_{g}^{m}}.$$

And hence,


(2) $$\;\;N_{g}^{X}=1/(2P_{X}(G=g|G>g-1))=\frac{9N_{g}^{f}N_{g}^{m}}{2N_{g}^{f}+4N_{g}^{m}}.$$

For comparison, on the autosomes, by the same reasoning but with half of the meioses from each sex and with diploid males,


$$P_{A}(G=g|G>g-1)=\frac{{\left(1/2\right)}^{2}}{2N_{g}^{f}}+\frac{{\left(1/2\right)}^{2}}{2N_{g}^{m}}.$$

And hence,


(3) $$N_{g}^{A}=\frac{1}{2}/\left(\frac{{\left(1/2\right)}^{2}}{2N_{g}^{f}}+\frac{{\left(1/2\right)}^{2}}{2N_{g}^{m}}\right)=\frac{4N_{g}^{f}N_{g}^{m}}{N_{g}^{f}+N_{g}^{m}}.$$

From Equations 2 and 3, the ratio of X to autosomal effective population sizes, which we denote as α, is


(4) $$\alpha =\frac{N_{g}^{X}}{N_{g}^{A}}=\frac{9(N_{g}^{f}+N_{g}^{m})}{8(N_{g}^{f}+2N_{g}^{m})}=\frac{9(1+(N_{g}^{m}/N_{g}^{f}))}{8(1+2(N_{g}^{m}/N_{g}^{f}))}.$$

Thus, α→9/8 as $N_{g}^{m}/N_{g}^{f}\to 0$, and α→9/16 as $N_{g}^{m}/N_{g}^{f}\to \infty$. Since α is a decreasing function of $N_{g}^{m}/N_{g}^{f}$, the ratio of X to autosomal effective population sizes satisfies 9/16<α<9/8.

With algebra, it can be shown that Equations 2 and 3 imply that


(5) $$N_{g}^{f}=\frac{2N_{g}^{X}N_{g}^{A}}{9N_{g}^{A}-8N_{g}^{X}}$$

and


(6) $$N_{g}^{m}=\frac{2N_{g}^{X}N_{g}^{A}}{16N_{g}^{X}-9N_{g}^{A}}.$$

Thus, given estimates of $N_{g}^{X}$ and $N_{g}^{A}$, one can use Equations 5 and 6 to obtain estimates for $N_{g}^{f}$ and $N_{g}^{m}$. These are standard equations for estimating sex-specific effective population sizes based on $N_{g}^{X}$ and $N_{g}^{A}$ (Wright 1969; Hartl and Clark 1997), although usually presented in the context of constant effective population sizes across time.

According to Equation 4, the allowable range of the X chromosome $N_{e}$ is between 9/16 and 9/8 of the autosome $N_{e}$ at each generation. When the X chromosome $N_{e}$ is overestimated or the autosome $N_{e}$ is underestimated, the estimated female effective population size (Equation 5) can be negative. Similarly, underestimation of the X chromosome $N_{e}$ or overestimation of the autosome $N_{e}$ can result in a negative estimate of the male effective population size (Equation 6).

### Analysis pipeline

We start with phased sequence data (using the true phase for simulated data and the inferred phase for real data), with males coded as haploid on the X chromosome. We use hap-ibd (Zhou *et al.* 2020) to infer IBD segments. For hap-ibd analysis on sequence data, we set the minimum seed length to 0.5 cM and the minimum extension length to 0.2 cM. The relatively small minimum seed length and minimum extension length increase the power to detect short IBD segments. We exclude rare variants by setting the minimum minor allele count filter to 100 because these lower-frequency variants are less informative, have less accurate phasing, and may be recent mutations.

We then run IBDNe on the detected IBD segments, with one analysis for autosomes and a separate analysis for the X chromosome. The genetic map file for the analysis is assumed to be a sex-averaged map. For the X chromosome, this means multiplying cM positions in the female-specific map by 2/3.

When applying IBDNe on simulated data (autosomes or X), we set “filtersamples = false” and “gmin = 1” because the chromosomes are simulated independently so that a pair of individuals can share ancestry 1 generation back (i.e. be siblings) on one chromosome without such sharing occurring on other chromosomes. These settings tell IBDNe not to look for and remove close relatives and to model IBD from shared ancestry starting from 1 generation before present.

Real data often have an excess of close relatives due to the sampling scheme. Thus, in the analysis of real autosomal data, we allow IBDNe to detect and remove close relatives, which is the default behavior. The X chromosome on its own is not sufficient to detect close relatives, so we use either available pedigree information or close relative pairs identified by IBDNe in the autosomal analysis to manually remove IBD segments from close relatives in X chromosome data. We then update the “npairs” parameter accordingly (see below) and set “filtersamples = false” in the X chromosome IBDNe analysis.

In the IBDNe analysis of X chromosome data, we set the “npairs” parameter equal to the number of haplotype pairs for which IBD segments could be present (i.e. all pairs except for those for which we have explicitly removed IBD segments). We first calculate the number of haplotype pairs based on the numbers of females and males in the sample using Equation 1. We then adjust the number of haplotype pairs to account for the number of close relative pairs that were removed. Removing a male–male pair decreases the count by 1. Removing a male–female pair decreases the count by 2. Removing a female–female pair decreases the count by 4.

In order for IBDNe to obtain bootstrap confidence intervals for the X chromosome effective population size estimates, we divide the X chromosome into 6 pieces of equal cM length. We recode the chromosome field of the IBD segment file using integer values between 1 and 6 according to the location of the IBD segments. IBD segments that cross more than 1 of these “chromosomes” are split into subsegments at the boundaries of the corresponding “chromosomes.”

After obtaining the X chromosome and autosomal effective population sizes, we estimate the female and male effective population sizes using Equations 5 and 6. We obtain bootstrap values for these estimates by taking pairs of bootstrap values from the X and autosomal analyses. For example, for the *n*th bootstrap value of the female effective population size at generation *g*, we take the *n*th bootstrap value for the X chromosome effective population size at generation *g* and the *n*th bootstrap value for the autosomal effective population size at generation *g* and apply Equation 5. After obtaining all the bootstrap values, we use the 2.5th and 97.5th percentiles to obtain an approximate 95% confidence interval for the female (for example) effective population size at generation *g*.

### Simulation study

We conducted a simulation study to evaluate the performance of our method. We used SLiM, a forward simulator (Haller and Messer 2019), to simulate the demographic history for the most recent 5,000 generations, and we used msprime, a coalescent simulator, to complete the simulation back to full coalescence of the sample (Kelleher *et al.* 2016; Haller *et al.* 2019). For all scenarios, we simulated data for 30 autosomes of length 100 Mb (Megabases) and an X chromosome of length 180 Mb.

For the simulation in SLiM, we used a mutation rate of $10^{-8}$ per base pair per generation and a recombination rate of $10^{-8}$ per base pair per meiosis on the autosomes and for female meioses on the X chromosome. We set the gene conversion initiation rate to $2\times 10^{-8}$ per base pair per meiosis on the autosomes and per female meiosis on the X chromosome, with a mean gene conversion tract length of 300 bp. We simulated populations with an equal sex ratio, and we also simulated populations with 20% females, 40% females, 60% females, and 80% females. We used the same total effective population size $\left(N_{g}^{f}+N_{g}^{m}\right)$ in each simulation. We sampled 50,000 individuals comprising 25,000 females and 25,000 males for each analysis.

We simulated data under a demographic model with a 4-stage exponentially growing population with an increased growth rate over time, which we call the “UK-like” scenario because it approximates the demographic history of the UK population (Browning and Browning 2013). The forward simulation of this model in SLiM starts from 5,000 generations ago with an initial size of 3,000. At 300 generations ago, this population starts to grow at an exponential rate of 1.4% per generation. At 60 generations ago, the rate of exponential growth increases to 6%. In the most recent 10 generations, the growth rate further increases to 25%, and the population size reaches around 21 million at the time of sampling.

The part of the simulation conducted in msprime corresponds to the demographic history earlier than 5,000 generations ago that has a constant population size of 3,000. When completing the simulations with msprime, we did not apply the gene conversion or sex-specific population size. In the msprime simulations, mutation occurred at a rate of $10^{-8}$ per base pair per generation and recombination at a rate of $10^{-8}$ per base pair per meiosis. There is no differentiation between female and male meioses in the generations prior to the starting generation of the forward simulation because msprime does not have sex-specific functionality. This lack of sex-specific treatment in the period more than 5,000 generations ago will affect the level of variation in the data but will not affect the distribution of IBD segments of length > 2 cM (the segments analyzed by IBDNe) because such segments typically have ancestry within the past 300 generations.

### Analysis of human populations

We applied the above analysis pipeline to whole-genome sequence data from the UK Biobank (Bycroft *et al.* 2018). The UK Biobank is a large-scale biomedical database that contains in-depth genetic, physical, and health data collected between 2006 and 2010 on half a million UK participants aged between 40 and 69 (Fry *et al.* 2017). The UK Biobank whole-genome sequencing (WGS) consortium recently released high-coverage whole-genome sequence data for 200,031 study participants (Halldorsson *et al.* 2022). We used Beagle 5.4 (Browning *et al.* 2021) and our previously described UK Biobank phasing pipeline (Browning and Browning 2023) to phase the 200,031 genomes on the UK Biobank Research Analysis Platform. We used 2 cM as the IBD length threshold and estimated the effective population size over the past 100 generations. We used the deCODE map in the analyses (Halldorsson *et al.* 2019).

We first analyzed the White British participants, who form the largest ethnic group in the UK Biobank. The UK Biobank sequence data include 91,532 White British females and 75,298 White British males. Since IBDNe has limits on the number of IBD segments that it can process, we randomly selected 5,000 White British females and 5,000 White British males for estimation of the autosome $N_{e}$. For analysis of the X chromosome $N_{e}$, we removed 16,234 randomly selected females to ensure equal numbers of females and males in the sample. For the autosomes, IBDNe removed close relatives using default settings. For the X chromosome, we used the UK Biobank's kinship estimates to identify and remove IBD segments from sibling pairs and parent–offspring pairs (Bycroft *et al.* 2018).

The UK Biobank also includes participants from several ethnic minority groups including Black British, Indian, Pakistani, Asian, and Bangladeshi. Among these, we chose to analyze the effective population size of the Indian group, which has a large sample size, although we note that this group contains considerable diversity. There are sequence data for 1,258 males and 1,293 females with Indian ancestry in the UK Biobank. We removed 35 randomly selected females to achieve equal numbers of females and males for the subsequent analysis. By default, IBDNe automatically removes IBD segments from pairs of related individuals and generates a list of these related pairs. We manually removed X chromosome IBD segments for these pairs of related individuals prior to estimation of the X chromosome $N_{e}$ using IBDNe.

## Results

### Simulation study

We checked that the distribution of X chromosome IBD segments in female–female haplotype pairs, in female–male haplotype pairs, and in male–male haplotype pairs is consistent in all simulations (Supplementary Fig. 2). We compared the estimated X chromosome $N_{e}$ obtained by splitting the X chromosome into 6 pieces to enable bootstrapping with the $N_{e}$ estimated on the undivided X chromosome, and the results are consistent (Supplementary Fig. 3).

**Fig. 1.**
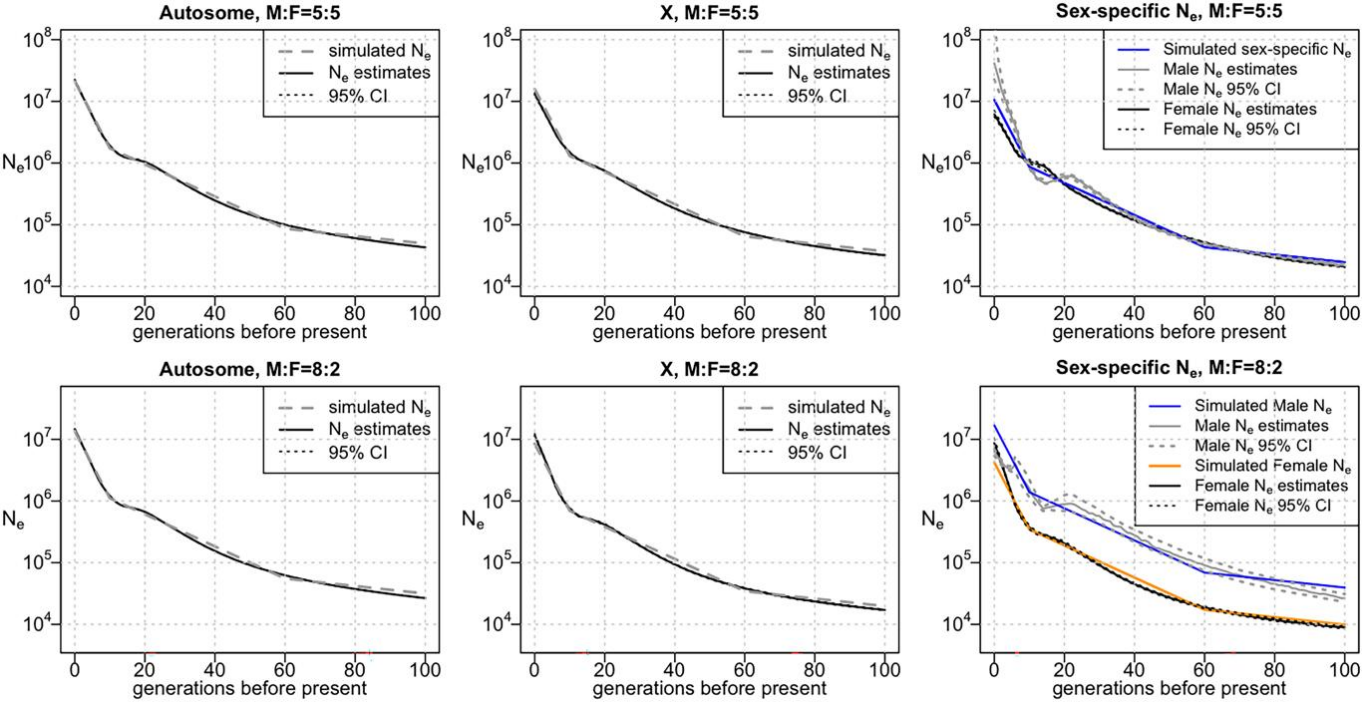
Estimates of the autosome, X chromosome, and sex-specific $N_{e}$ in a UK-like simulation study. Autosomal $N_{e}$ is shown in the left column, X chromosome $N_{e}$ in the middle column, and sex-specific $N_{e}$ in the right column. The top row is with an equal sex ratio, while the second row is with 20% females. Results for other choices of sex ratios can be found in Supplementary Fig. 4. *Y*-axes show $N_{e}$ plotted on a log scale. In cases where the estimated sex-specific $N_{e}$ is negative (see *Methods*), it is not shown.

We compared $N_{e}$ estimated from the simulated autosome and X chromosome data to the actual $N_{e}$ for the UK-like demographic model. The actual autosome or X chromosome $N_{e}$ can be obtained from the sex-specific effective population sizes using Equations 2 and 3. The estimated $N_{e}$ generally matches the true $N_{e}$ closely (Fig. 1 and Supplementary Fig. 4). However, some discrepancies exist between the estimated and actual $N_{e}$ because IBDNe cannot localize sharp changes in the population size to the exact generation and it tends to oversmooth corners of the trajectory of the effective population size over time (Browning and Browning 2015).

We next used the estimated X chromosome $N_{e}$ and autosome $N_{e}$ to estimate the sex-specific effective population sizes. We find that the formulas for the sex-specific $N_{e}$ as functions of the autosome and X chromosome $N_{e}$ (Equations 5, 6) are sensitive to errors in $N_{e}$ estimation. The estimated sex-specific $N_{e}$ is similar to the actual values most of the time, but at around 15 generations ago, where there was a large change in the population growth rate, we observe greater inaccuracy in the estimated $N_{e}$, and the estimated sex-specific $N_{e}$ differs from the actual sex-specific $N_{e}$ (Fig. 1 and Supplementary Fig. 4).

### UK Biobank data

**Fig. 2.**
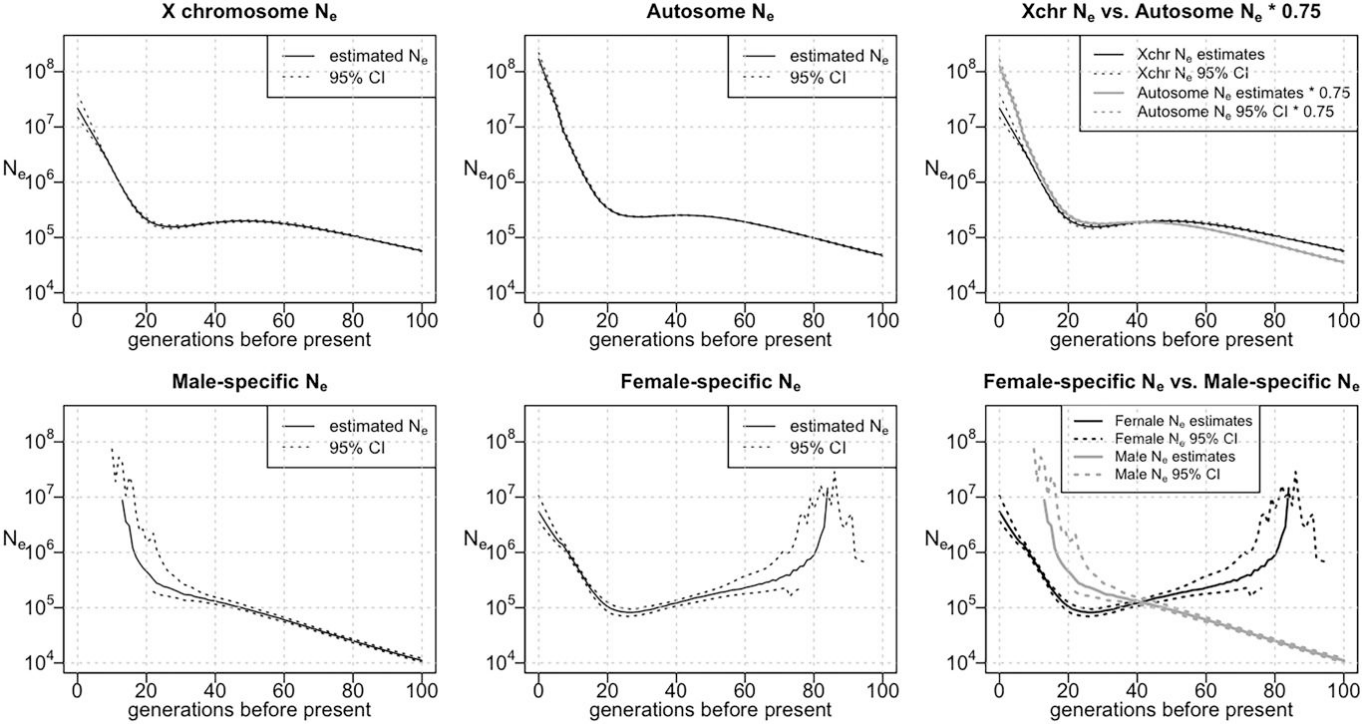
Effective population size of the UK Biobank White British group. From left to right, the top row shows the estimated X chromosome $N_{e}$, the estimated autosome $N_{e}$, and a comparison of the estimated X chromosome $N_{e}$ with 75% of the estimated autosome $N_{e}$. The bottom row displays the male-specific $N_{e}$, the female-specific $N_{e}$, and a comparison between them. For the $N_{e}$ plots, the *Y*-axes show $N_{e}$ on a log scale. In cases where the estimated sex-specific $N_{e}$ or its confidence band is negative (see *Methods*), the negative values are not shown.

**Fig. 3.**
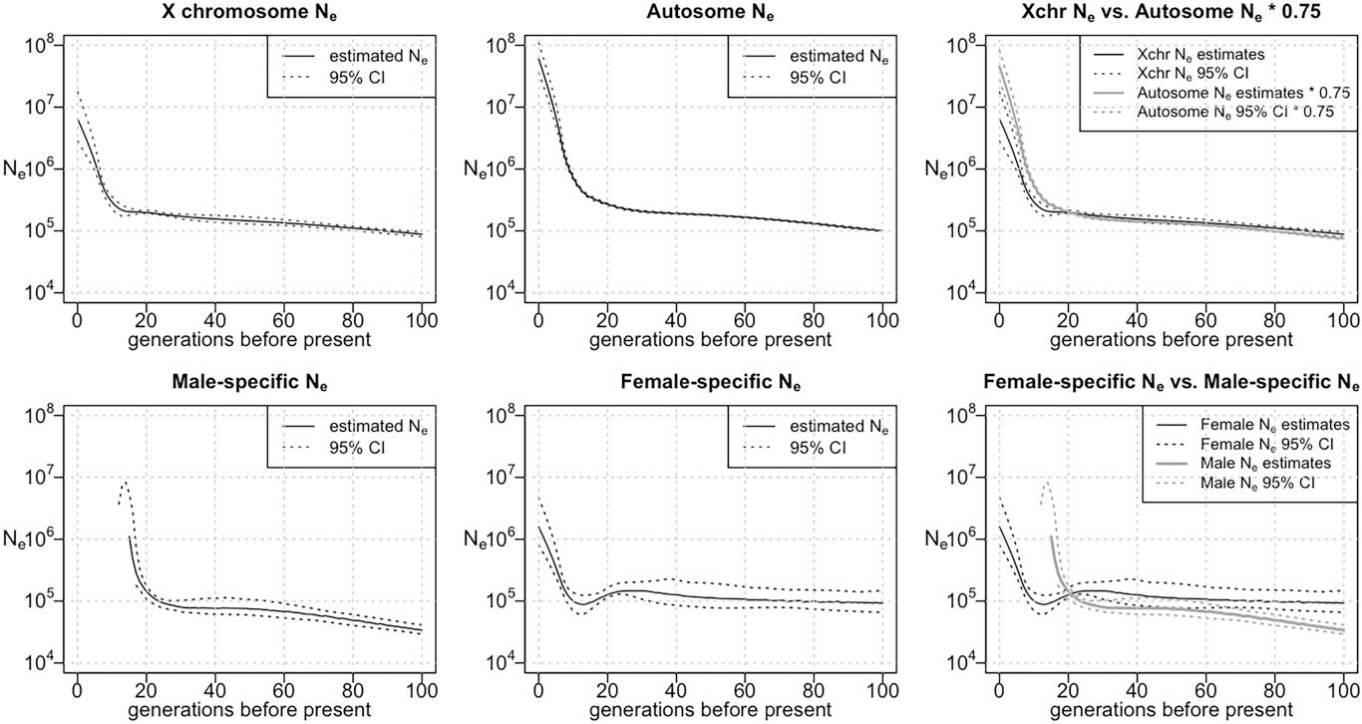
Effective population size of the UK Biobank Indian group. From left to right, the top row shows the estimated X chromosome $N_{e}$, the estimated autosome $N_{e}$, and a comparison of the estimated X chromosome $N_{e}$ with 75% of the estimated autosome $N_{e}$. The bottom row displays the male-specific $N_{e}$, the female-specific $N_{e}$, and a comparison between them. For the $N_{e}$ plots, the *Y*-axes show $N_{e}$ on a log scale. In cases where the estimated sex-specific $N_{e}$ or its confidence band is negative (see *Methods*), the negative values are not shown.

The estimated autosome $N_{e}$ for the UK Biobank White British population (Fig. 2) went through a period of moderate growth between 50 and 100 generations ago. Between 20 and 50 generations ago, the effective population size was fairly constant. The effective population size had a high rate of growth in the most recent 20 generations and reached a current population size of 169 million (95% confidence interval = 139–221 million). IBDNe estimates the most recent generations by extrapolating the growth rate of earlier generations and does not account for a possible recent decrease in the population growth rate; hence, the $N_{e}$ for generation 0 may be overestimated (Browning and Browning 2015). The autosome $N_{e}$ estimated from the genotype data of the UK Biobank Indian participants (Fig. 3) shows slow growth from 100 generations ago until around 10 generations ago and a higher rate of growth in the past 10 generations. The estimated current effective population size is 89 million (95% confidence interval = 47–221 million).

The inferred X chromosome $N_{e}$ has a similar shape to the inferred autosome $N_{e}$ for both groups. Notably, the estimated X chromosome $N_{e}$ is fairly close to 75% of the inferred autosomal effective population size, which is the expected effective population size that would be obtained from the X chromosome data if the female and male effective population sizes have been equal in these populations (Figs. 2 and 3). However, the estimated 75% autosome effective size is lower than the X chromosome effective size between 42 and 100 generations ago for the UK Biobank White British population, while the estimated 75% autosome effective size is higher than the X chromosome effective size in the past 41 generations, although the confidence intervals for the 2 estimates overlap in parts of this range. In the UK Biobank Indian group, we see a similar pattern, with a higher estimated X chromosome size between 22 and 100 generations ago and a higher estimated 75% autosome effective size in the past 21 generations, although in the period between 22 and 100 generations ago, the estimates are very close and the confidence intervals largely overlap.

To investigate the historical sex-specific effective population sizes of the UK Biobank White British population and the UK Biobank Indian population, we used the X chromosome and autosome $N_{e}$ to estimate the female and male $N_{e}$ of these 2 populations. For the UK Biobank White British population, there is an apparent dip in the female effective size around 27 generations ago (Fig. 2, bottom center), but this may be an artifact similar to the dip in the estimated effective size in the simulated UK-like population around 15 generations ago (Fig. 1, top right); both of these dips occur around the timing of an increase in the population growth rate. For the Indian group, a similar dip appears in the estimated female effective size around 13 generations ago. For both groups, ignoring generations for which estimates are negative, we find that in the more recent past (up to 41 generations ago for the UK Biobank White British group and up to 21 generations ago for the UK Biobank Indian group), the estimated male effective population size is higher than the female, while this is reversed in the more distant past.

The sex-specific $N_{e}$ estimates for both the White British group and the Indian group contain negative values. The estimated female effective population size of the White British group is negative earlier than 86 generations before present due to overly high X chromosome $N_{e}$ estimates compared to autosome $N_{e}$ estimates. Likewise, the estimated male effective population size of this group is negative from 13 generations ago to the present as the X chromosome $N_{e}$ estimates are low compared to autosome $N_{e}$ estimates. In addition, the estimated male effective population size of the Indian group is negative from 13 generations ago to the present as a result of excessively low X chromosome $N_{e}$ estimates compared to autosome $N_{e}$ estimates. The negative $N_{e}$ estimates over these periods are not scientifically meaningful and are generally accompanied by large confidence intervals. For both the White British group and the Indian group, the distribution of X chromosome IBD lengths is mostly consistent in male–male, female–male, or female–female haplotypes. The rate of IBD segments that are longer than 8 cM (sex-averaged unit) is slightly higher for male–male IBD haplotypes in the Indian group (Supplementary Fig. 2). However, the slight difference among rates of long IBD segments in the 3 categories is likely a consequence of sampling variability as discussed in the legend of Supplementary Fig. 2.

## Discussion

Previous studies have shown that the X chromosome can provide information about demographic processes that cannot be revealed by the analysis of autosomes alone (Ramachandran *et al.* 2004; Schaffner 2004; Bustamante and Ramachandran 2009; Bryc *et al.* 2010; Goldberg and Rosenberg 2015; Shringarpure *et al.* 2016). In this work, we focused on utilizing IBD segments on the X chromosome to infer the X chromosome effective population size. We developed a framework to model the X chromosome $N_{e}$ and derived the relationship between the X chromosome and autosome $N_{e}$ by considering the different coalescence rates between X chromosomes and between autosomes. We also showed how to apply this information to calculate the female and male effective population sizes in a population as functions of the X chromosome and autosome $N_{e}$.

We applied our method to estimate the X chromosome effective population size for the UK Biobank White British individuals and the UK Biobank Indian individuals. In both populations, we observe a time point within the past decades of generations when the male estimated $N_{e}$ intersected with and continued to exceed the female estimated $N_{e}$. We speculate that demographic trends such as polygamy that favored a lower male effective population size may have been more prevalent in the more distant past and that migration rates, which have increased in recent times, may have increased more in males than in females, which would increase the male effective population size relative to the female effective population size. However, it is also possible that artifacts such as better detection of long IBD segments on the X chromosome due to more accurate phasing resulting from the smaller effective population size and haploid males could be responsible for these trends.

We validated the performance of our method in a simulated population with similar growth rates and IBD rates as the UK population. We found that the estimated sex-specific effective population sizes capture the overall trends of the true female and male effective population sizes. In the simulations, however, we observe some significant discrepancies between the estimated sex-specific $N_{e}$ and the true sex-specific $N_{e}$ across timescales of tens of generations, which tends to occur around points in time at which the population growth rate changed. This indicates that the estimated sex-specific $N_{e}$ calculated from the estimated autosomal and X chromosome $N_{e}$ should not be used to test hypotheses about differences in the effective population size between sexes, particularly when those hypotheses involve differences that occur for limited time periods rather than across the full estimation timescale.

Although X chromosome IBD information has been used by previous studies for the estimation of genealogical relations between individuals, especially for kinship estimation in forensic settings (Pinto *et al.* 2011; Buffalo *et al.* 2016; Henden *et al.* 2016), there has been a lack of studies that use X chromosome IBD segments to estimate the recent effective population size. Our work thus fills a gap that existed in the application of X chromosome IBD information in population genetic studies.

Previous methods for estimating sex-specific population history or sex bias in human populations have relied on comparisons of genetic diversity between autosomes and the X chromosome using allele frequency differentiation, patterns of neutral polymorphism, and the site frequency spectrum (Ramachandran *et al.* 2004; Hammer *et al.* 2008; Keinan *et al.* 2009; Emery *et al.* 2010; Clemente *et al.* 2018; Musharoff *et al.* 2019). Most of these methods considered only a single estimate of the effective sex ratio over the entire history of a population, although this ratio can vary over time (Hammer *et al.* 2008; Keinan *et al.* 2009; Emery *et al.* 2010; Clemente *et al.* 2018). Some of these methods also focused on a constant overall effective population size across time, although changes in the effective population size can distort these analyses (Ramachandran *et al.* 2004; Keinan *et al.* 2009). Recently, Musharoff *et al.* (2019) developed a likelihood ratio test for population sex bias that considered populations of nonconstant size and changing sex ratios using site frequency spectrum data. However, this method requires demographic parameters to be constant within time epochs. In comparison, our approach for estimating the X chromosome effective population size and the sex-specific effective population sizes requires minimal assumptions and allows the effective population sizes to vary independently over time. The ability of our IBD-based analyses to infer effective population sizes in the past hundred generations distinguishes our approach from other methods.

There are several limitations to our approach. First, IBD-based estimation of the effective population size requires a large sample of individuals from the population. The performance of IBDNe is affected by the number of detected IBD segments. For example, we observe wider confidence bands for the estimated $N_{e}$ in the most recent generations since there tend to be fewer very long IBD segments in the sample. Similarly, we observe wider confidence bands for the X chromosome $N_{e}$ compared to the autosomal $N_{e}$ due to the smaller amount of data in the X chromosome.

Second, our method for estimating $N_{e}$ is less accurate around times when the population experienced an abrupt change in the population size or growth rate. It tends to oversmooth the autosomal and X chromosome trajectories, which can then induce spurious oscillations in the estimated sex-specific effective population size over time. Moreover, these artifacts persist in the bootstrapped data so that the bootstrap confidence intervals are overconfident in such regions and do not have the desired level of coverage of the underlying effective population sizes. Given these limitations, we note that although our method provides reliable estimates of the X chromosome effective population size in simulated data, its application to estimate the sex-specific population history is not suitable for rigorously testing hypotheses on the sex-specific $N_{e}$ in a population because small inaccuracies in estimation of the autosome and X chromosome effective population sizes are magnified when transforming these to estimates of the sex-specific effective population size. We thus recommend only considering the estimated sex-specific effective population size as a tool to explore the overall pattern of sex-specific past demographic events.

## Supplementary Material

jkad165_Supplementary_Data

## Data Availability

UK Biobank data were obtained via the UK Biobank Research Analysis Platform. The IBDNe software is available from https://faculty.washington.edu/browning/ibdne.html. The hap-ibd software is available from https://github.com/browning-lab/hap-ibd. Supplemental material are available at G3 online.
