# Supplementary material for: Identity-by-descent-based estimation of the X chromosome effective population size with application to sex-specific demographic history
Source: G3 (Bethesda). 2023 Jul 27;13(10):jkad165. doi: 10.1093/g3journal/jkad165 (PMC10542559; doi:10.1093/g3journal/jkad165)
Supplement: jkad165_Supplementary_Data [file jkad165_supplementary_data.docx]

# Supplementary Figures

**Figure S1. Simulated distribution of the length of an X chromosome IBD segment measured in sex-averaged genetic distance given the number of generations to the shared ancestor.** The distribution of 10,000 simulated $\text{exponential}(3F/2)$ observations, where $F\sim\text{binomial}(2g, 2/3)$, is compared to the $\text{exponential}(2g)$ density, shown as the red curve in each plot, for $g=1, 2, 5$ and $100$ generations. The simulated distribution is for the distribution of the length of the segment when assuming Haldane’s model, while the $\text{exponential}(2g)$ is the distribution used in our methodology. When $F=0$, the simulated length of IBD segment is set to 100 Morgans. Although not shown in the figure, the observations corresponding to $F=0$ were accounted for when calculating the simulated density.

**
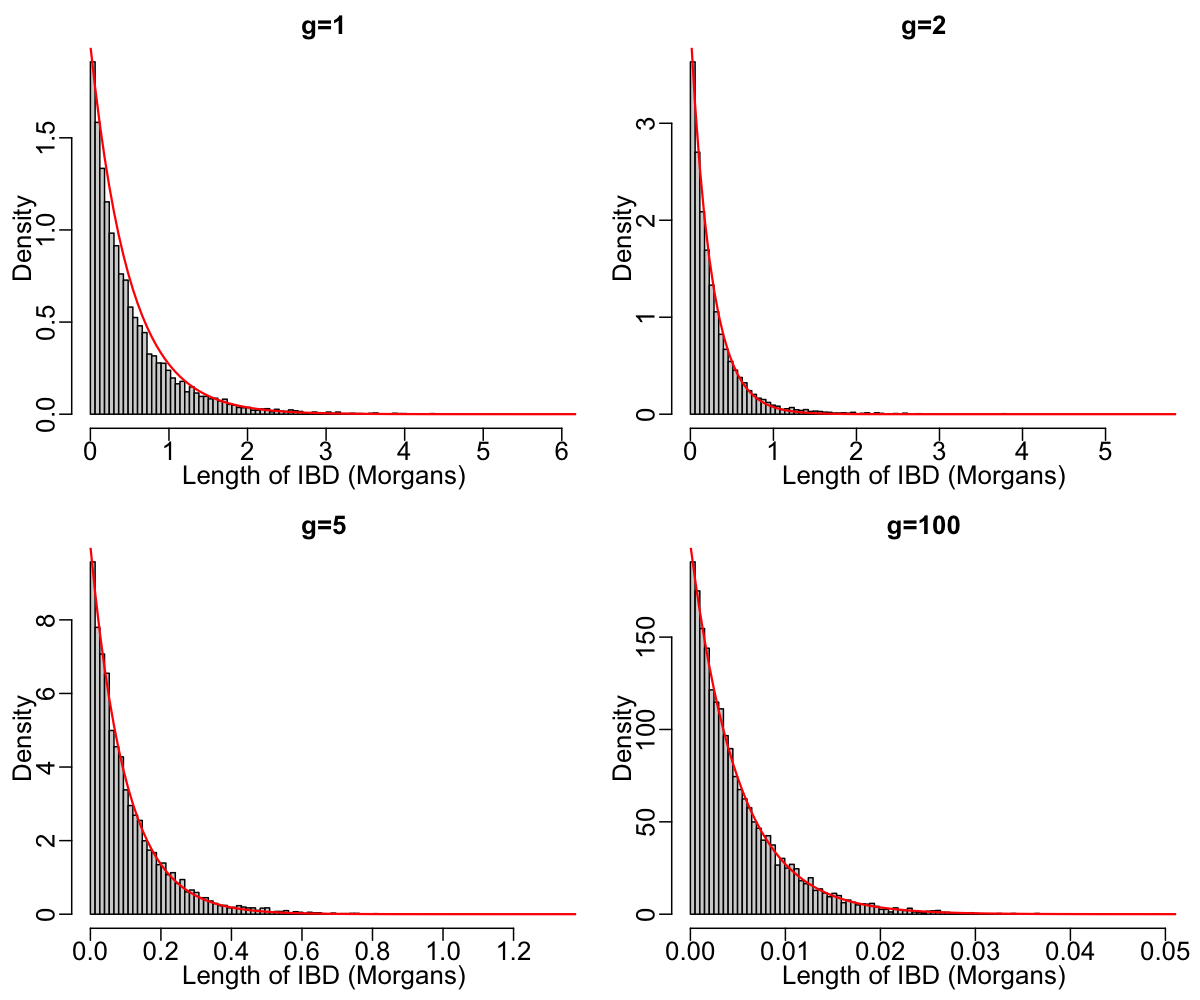
**

**Figure S2. The distribution of X chromosome IBD segments between sexes.** The black line, red line and green line show the rate of IBD segments on a log scale over a series of consecutive length bins in female-female (ff) haplotype pairs, male-male (mm) haplotype pairs, and male-female (mf) haplotype pairs, respectively. The rate of IBD segments in each length bin for each sex combination is calculated as the number of IBD segments from pairs of individuals with the corresponding sex in that bin divided by the total number of haplotype pairs of the corresponding sex combination. IBD lengths are measured in sex-averaged units. The left column displays results from the UK-like simulation with equal sex ratio. The middle column displays results from the White British group in the UK Biobank sequence data. The right column displays results from the Indian group in the UK Biobank sequence data. There is a slightly higher rate of male-male IBD segments that are longer than 8 cM (sex-averaged unit) in the Indian group, but this difference may not be significant given the small number of IBD segments in the three categories (204 in female-female haplotypes, 71 in male-male haplotypes, and 205 in male-female haplotypes). Since the number of IBD segments with length in this range are approximately Poisson distributed, the magnitude of the difference observed between the count of male-male haplotypes and female-female or female-male haplotypes is about 2x pooled standard deviations.

**
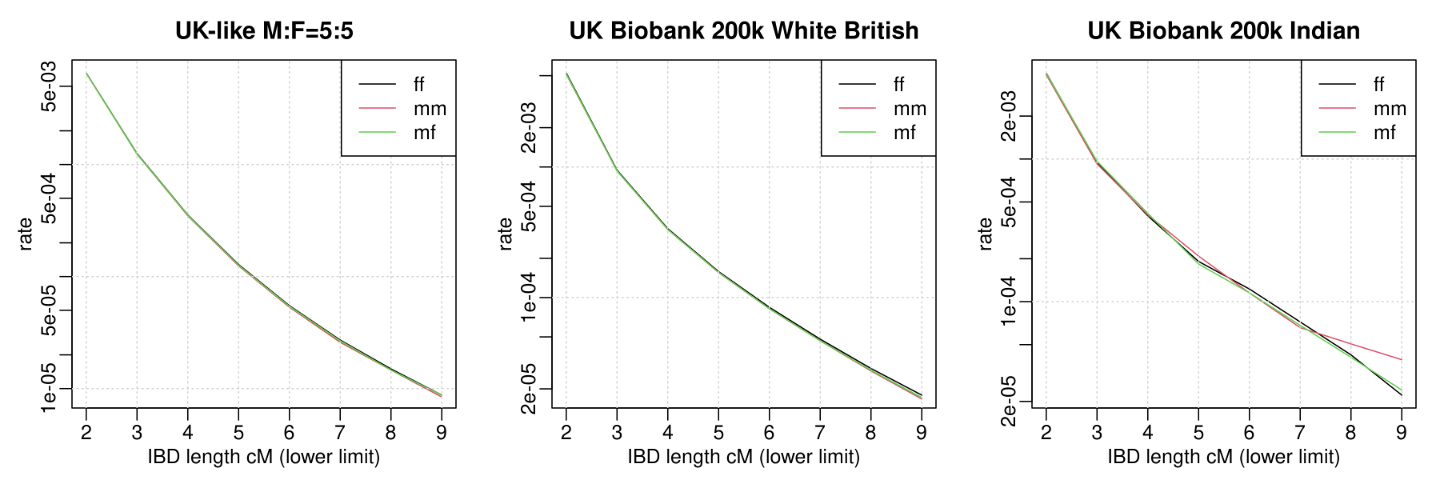
**

**Figure S3. Effective population size estimated from an undivided X chromosome versus the effective population size estimated by splitting the X chromosome into six regions.** $N_{e}$ estimated on a single undivided X chromosome is shown in the left column. $N_{e}$ estimated by treating six separate regions of the X chromosome as six chromosomes to enable bootstrapping is shown in the right column. In each plot, the Y-axes are on a log scale. From top to bottom, the rows display results from a UK-like simulation with equal sex ratio, the White British group in UK Biobank, and the Indian group in UK Biobank.

**
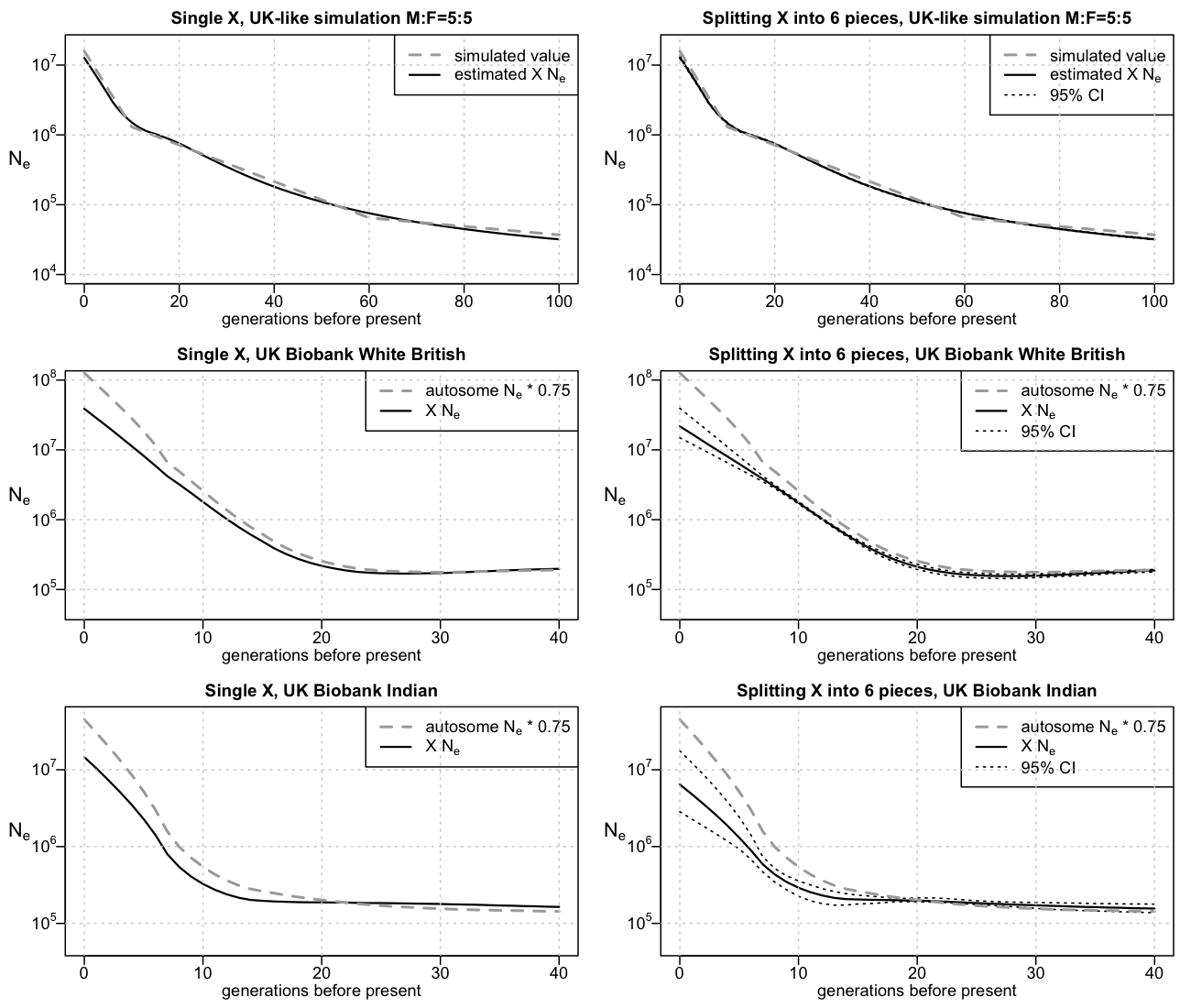
**

**Figure S4. Estimates of autosome, X chromosome, and sex-specific** $\boldsymbol{N}_{\boldsymbol{e}}$ **in UK-like simulations.** Autosomal $N_{e}$ is shown in the left column, X chromosome $N_{e}$ in the middle column, and sex-specific $N_{e}$ in the right column. From top to bottom, the rows display results from a UK-like simulation with 80%, 60%, and 40% females. The Y-axes are on a log scale.

**
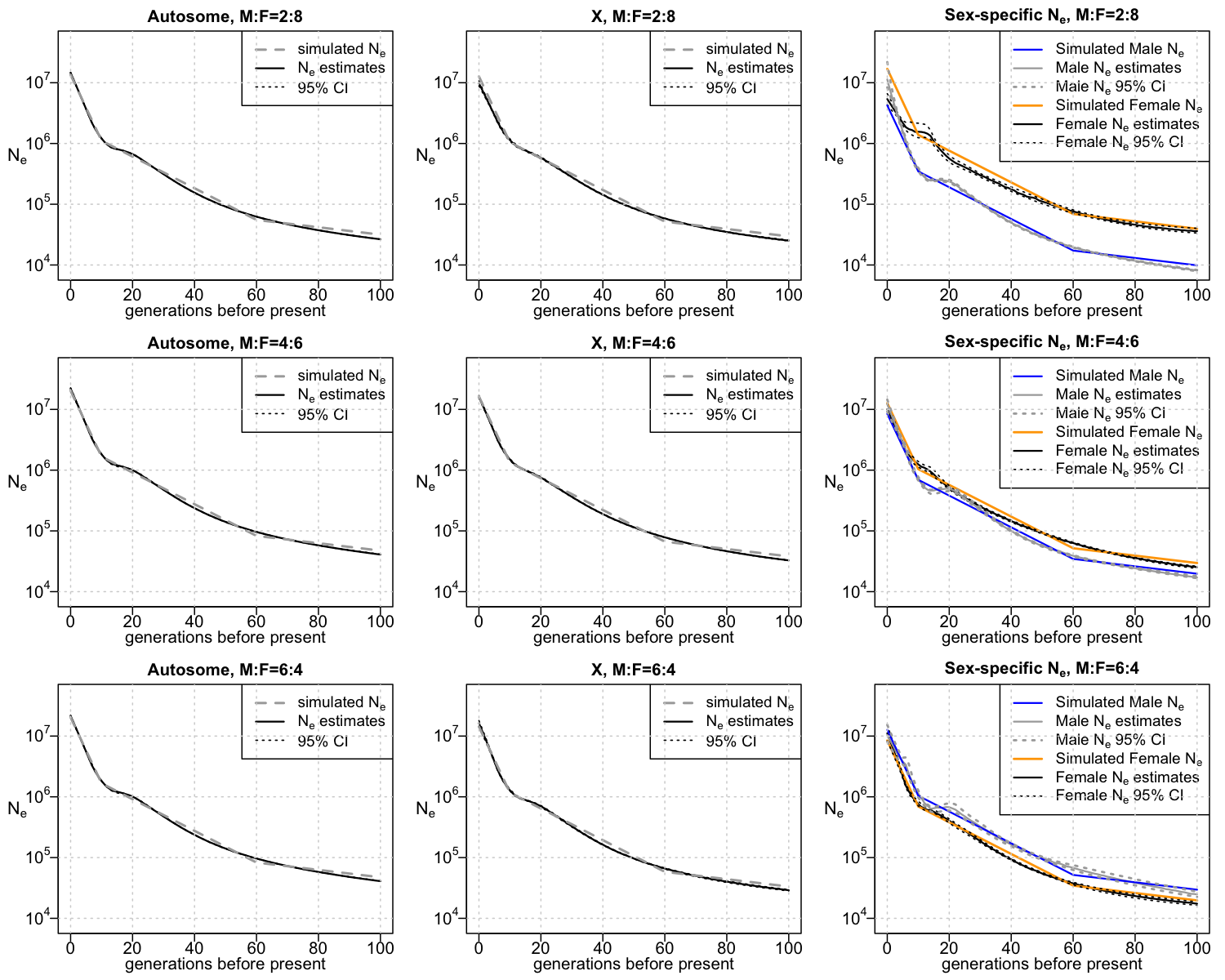
**
